# Sustainable moisture-driven electricity generation using waste materials

**DOI:** 10.1038/s41598-026-61180-8

**Published:** 2026-07-14

**Authors:** Aman Ul Azam Khan, Nazmunnahar Nazmunnahar, Mortuza Hasan, Abdul Baqui

**Affiliations:** 1 Department of Textile Engineering, BGMEA University of Fashion and Technology, Dhaka, 1230 Bangladesh; 2 Department of Earth and Environment Science, The University of Manchester, Manchester, M13 9PL UK; 3 School of Engineering and Technology, Central Queensland University (CQU), Melbourne, VIC Australia

**Keywords:** Energy science and technology, Engineering, Environmental sciences, Materials science

## Abstract

Atmospheric moisture is an abundant, renewable resource with potential for sustainable energy harvesting. While moisture-material interactions can generate electricity under ambient conditions, most current systems remain expensive, and produce voltages too low for direct use in wearable electronics. To address these limitations, we developed a moisture-driven electric generator (MEG) from waste biomass and recycled materials that convert ambient humidity into electrical output. The MEG integrates wild sugarcane fibers and recycled cigarette-butt cellulose with an upcycled carbon-paste layer, which enhances moisture uptake, ion dissociation, and directional ion migration across asymmetric current collectors. A single unit delivers up to 1.16 V and 16.44 µW cm^−3^, operates under ambient humidity conditions, and restores voltage after drying following renewed natural moisture reabsorption. A basic conceptual model is proposed in which moisture adsorption within the hygroscopic composite promotes NaCl ion dissociation and directional ionic transport, leading to interfacial charge separation between asymmetric electrodes and the generation of a measurable potential difference. Scalable series/parallel configurations boost voltage and current, enabling direct operation of low-power electronic devices under maintained humid conditions without external capacitors. This low-cost approach highlights moisture-activated textile composites as sustainable power sources for self-powered and low-power electronic systems.

**Supplementary Information:**

The online version contains supplementary material available at https://doi.org/10.1038/s41598-026-61180-8.

## Introduction

Water is a sustainable and recyclable resource that plays a pivotal role in Earth’s energy dynamics through the natural hydrologic cycle. While energy harvesting from liquid water has been explored extensively, the vast potential of atmospheric moisture remains underutilized^1,2^. Hydrovoltaic power generation enables energy production by harvesting atmospheric water vapor in its gaseous state^3^. It directly generates electricity^4^ via water-material interactions, provides enhanced flexibility, and complements conventional renewable energy sources^5^.

The early development of hydrovoltaic power generation has progressed rapidly with a particular focus on droplets^6^, waves^7^, and water evaporation^8^. Moisture is a key component of the natural water cycle that acts as a medium for transferring thermal, mechanical, and chemical energy^9^. The use of low-dimensional nanostructures for harvesting moisture-induced energy presents a promising strategy to meet future energy demands through enhanced ionization and rapid ion transport^10^. Even recent investigations into moisture-material interactions have led to significant advancements in fields such as water splitting^11^. Recent study has employed hygroscopic hydrogels as water-absorbing electrolytes in conjunction with photoelectrochemical catalysts to facilitate moisture splitting^12,13^. Significant advancements have been made across emerging fields and applications^14–17^. For instance, optimizing the site-specific water binding properties of metal-organic frameworks (MOFs) has notably enhanced the efficiency of atmospheric water harvesting (AWH)^18^.

Notably, moisture-driven electricity generation (MEG) has attracted significant attention in the past few years^2^. It utilizes streaming currents^4,19–21^ or ion drift driven by concentration gradients^22^ to convert the chemical energy of moisture into electrical energy. Many functional materials such as nanostructured carbon^4^, graphene^23^, carbon black^4^, metal oxide nanowires^24^, and proteins^25^ have been utilized in MEG systems to efficiently harvest electrical energy from moisture. Natural hydrophilic substances^26^ like lignocellulosic materials have emerged as valuable resources for constructing MEGs. Moreover, MEGs can be designed from organic or inorganic materials independently or through the integration of organic–inorganic composite nanomaterials^27–29^.

However, these technologies face significant challenges. A major concern is the high cost of materials in MEG development, which could restrict large-scale implementation and reduce accessibility for end users. Moreover, the requirement for partial sealing complicates fabrication and limits design flexibility, and the spacing required between current collectors to sustain the moisture gradient can further increase internal resistance^2^. Conversion occurs only during water sorption^30^; if an MEG material or device is left exposed to an open environment when not in use, it ultimately becomes incapable of power generation^2^. Moisture-electric generators (MEGs) typically produce voltages of ≤ 0.6 V, which is insufficient for direct integration into wearable electronics and smart textile applications^31^.

Thus, these limitations constrain the widespread development and practical implementation of MEG materials and devices. To address these challenges, this study develops a MEG system using sustainable and low-cost materials, enabling greater scalability, simplified fabrication, and enhanced energy generation efficiency, thereby promoting the practical adoption of MEG technologies. Unlike electrochemical batteries, which rely on reversible redox reactions and internal chemical energy storage, MEGs produce electricity only in the presence of a humidity gradient. In such systems, voltage decay reflects equilibration of the internal moisture gradient rather than depletion of stored chemical energy. Accordingly, MEGs operate as gradient-driven energy harvesters rather than as energy storage devices.

## Materials and methodology

### Engineering a moisture-driven electric generator (MEG)

To engineer the MEG, the composite structure was strategically designed to enhance power generation. The current collector was created on the surface of the composite. This structure comprises multiple functional layers in a planar configuration that enhance moisture-driven ion transport and electrical output while ensuring flexibility.

### Architecting the composite

**Fig. 1:**
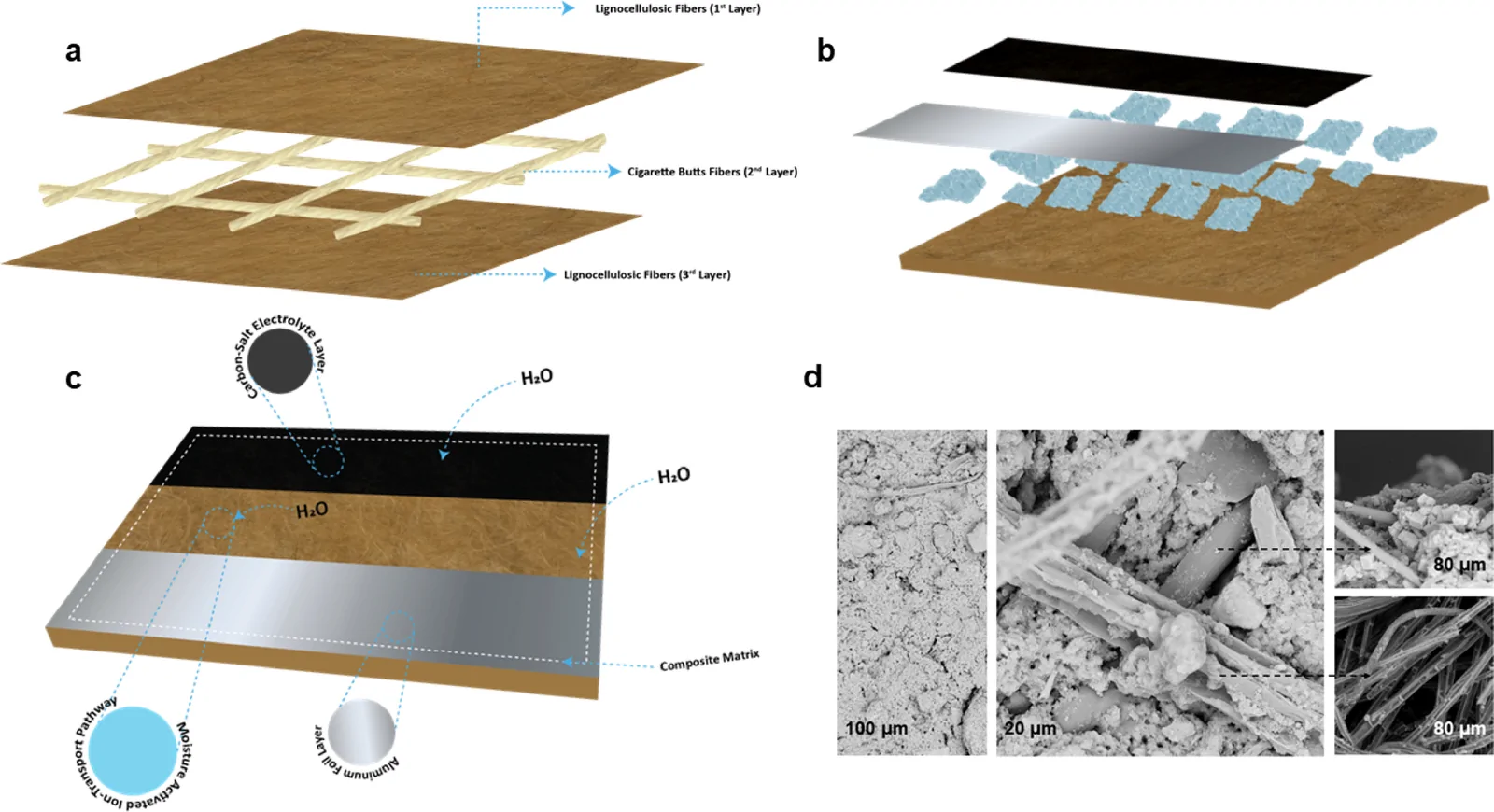
Design and structure of the MEG. (**a**) Schematic of the composite architecture comprising wild *Saccharum spontaneum* (Wild Sugarcane) and cigarette-butt fibers (CBF). (**b**) 3D symmetric representation of the structural sequence and fabrication of the MEG based composite. (**c**) 3D schematic overall structure of the MEG. (**d**) SEM micrographs of the coating surface and internal porous structure showing interwoven fibers (CBF and wild sugarcane) and carbon coating that facilitates moisture absorption and ion transport.

The design of the composite was illustrated in Fig. 1a. Lignocellulosic material such as *Saccharum spontaneum* L. (wild sugarcane or kans grass) was used for its relatively high moisture content and hydrophilic nature, which shows strong water affinity due to its polar structure.^32^ The plant material was identified as *Saccharum spontaneum* L. based on its morphological characteristics by Ms. Afrin Sultana Ivee (MSc, Department of Botany, University of Dhaka, Bangladesh), using standard taxonomic keys and authenticated regional floras for the genus *Saccharum*. Wild sugarcane was collected from naturally occurring populations in the Diabari area of Uttara, Dhaka, Bangladesh (23°51′25.6″N, 90°22′08.4″E) on 17 October 2023 during the post-monsoon season. The plants were collected from open grassland vegetation where *Saccharum spontaneum* grows naturally. Immediately after collection, the plant material was washed with water to remove adhering soil and debris, air-dried under ambient conditions, manually separated into fibers, and stored under laboratory conditions prior to composite fabrication. At the time of this study, a voucher specimen had not been deposited in a publicly accessible herbarium; therefore, a deposition number is not available. This affinity allows efficient moisture uptake from the environment^33^, which is necessary for MEG. Wild sugarcane, categorized as a waste material due to natural dispersion of mature stalks (Supplementary Fig. 1), minimizes the composite cost. It was mixed with cigarette butt fibers (CBF), also considered waste material. Cigarette butt fibers (CBF), composed primarily of cellulose acetate, are not inherently strongly hygroscopic; however, their fibrous and porous morphology provides a mechanically stable scaffold for anchoring salt and conductive fillers while facilitating efficient moisture diffusion throughout the composite. Fibers were extracted from cigarette butts following a series of defined steps (Supplementary Fig. 2). Cigarette butts were collected from bins, stores, and public spaces. They were manually cleaned of unburned tobacco and ash. The paper coating was removed with a blade, and the cellulose acetate filter was extracted and hand shredded. Fibers were washed in hot water (70 °C) for 30 min, then scoured and bleached with aqueous NaOH (99.9% purity) and H_2_O_2_ (50% concentration) at 95 °C for one hour^34^. Treated fibers were immersed in 0.02% H_2_SO_4_ (100 ml) for 30 min at room temperature^35^, then rinsed several times with cold water. The fibers were briefly rinsed with acetone at room temperature (< 2 min exposure) to remove surface contaminants, followed by thorough drying at 60 °C. The acetone rinse was applied only for surface cleaning, and the exposure time was strictly limited to prevent dissolution or structural alteration of the cellulose acetate fibers, which are known to be sensitive to prolonged acetone exposure^36^. These purified fibers were integrated into the composite structure.

A quadrilateral composite structure was fabricated by first preparing thin sheets from sequential layers of wild sugarcane fibers. A 100 °C boiled 0.95 ml saline solution (sea salt and water) was sprayed on the sheet. (Supplementary Fig. 3) Then, cigarette butt fibers were arranged in a grid pattern of horizontal and vertical lines to form squares. (Supplementary Fig. 4) Again 0.95 ml of saline solution was applied. The second thin sheet of wild sugarcane fibers was layered, followed by a final saline spray. Polyvinyl acetate resin ((C_4_H_6_O_2_)_n_) was used as a binder to ensure cohesion. The composite was processed into thin sheets by felting process, thoroughly dried, and cut into quadrilateral shapes.

### Fabrication of functional electrode layer and current collector for the MEG

Current collectors and electrode interfaces play a crucial role in enabling charge collection and transport in MEGs^37^. The positive electrode layer was fabricated on one side of the composite using a Carbon Black (CB)-Manganese Dioxide (MnO_2_) mixture sourced from non-functional dry cell batteries, classified as e-waste. Porous CB provides a high surface area that facilitates interfacial charge accumulation and ion adsorption, thereby promoting moisture-induced charge separation and transport^38^. MnO_2_ was incorporated as an interfacial additive that may contribute to charge buffering at the electrode interface^39^.

This CB-MnO_2_ mixture enables upcycling of waste materials into higher-value functional components. Sea salt commonly known as sodium chloride (NaCl) was selected for its environmental stability and its ability to absorb up to 500% of its weight in moisture under high relative humidity conditions^2^. Thus, NaCl absorbs moisture under high RH (Supplementary Fig. 5). Although other hygroscopic salts like lithium chloride (LiCl) and calcium chloride (CaCl_2_)^40^ were considered (Supplementary Fig. 6) but NaCl was selected for cost-effectiveness, high availability environmental safety, and optimal ionic activity. Moreover, experimental analysis further indicates that NaCl exhibits the fastest moisture uptake on the composites (Supplementary Fig. 7).

The mixture of Carbon Black, MnO_2_, sea salt, and water is referred to as the mixture-salt electrolyte solution. Owing to the limited solubility of the CB-MnO_2_ powder in saline water (Supplementary Fig. 8), a minimal amount of water was used to form a brushable paste. The solution was applied to one side of the composites by brush coating. The effective two-probe DC conductivity increased from 1.37 × 10^−7^ S m^−1^ at 5.41 g m^−2^ loading density to 1.0 × 10^−6^ S m^−1^ at 9.46 g m^−2^ after three coating cycles (Supplementary Figs. 9–10). These values are used only as comparative indicators of improved charge/ion transport within the coated composite and are not interpreted as intrinsic electronic conductivity. Coated composites were thoroughly dried in oven at 70 °C.

Experimental evidence demonstrates that the carbon-coated composite exhibits enhanced moisture absorption and desorption behaviour compared with the uncoated composite, which is essential for effective moisture-driven charge generation in MEG devices (Supplementary Fig. 11). Ultra-flexible aluminum foil collected from e-waste served as the negative current collector to ensure cost efficiency and sustainability. It was collected and washed with detergent at 60 °C for 30 min, then attached to the other side of the composite’s top surface using polyvinyl acetate. The entire structure was dried in oven at 70 °C. The entire fabrication of the MEG-based composite is shown in Fig. 1b, with a 3D schematic of the structural design presented in Fig. 1c. Further characterization using scanning electron microscopy (SEM) revealed the fibrous architecture, coated surface of the composite (Fig. 1d) and porous composition, while energy-dispersive X-ray spectroscopy (EDX) showed the elemental distribution of Na and Cl (Supplementary Fig. 12).

### Working principle of the MEG

**Fig. 2:**
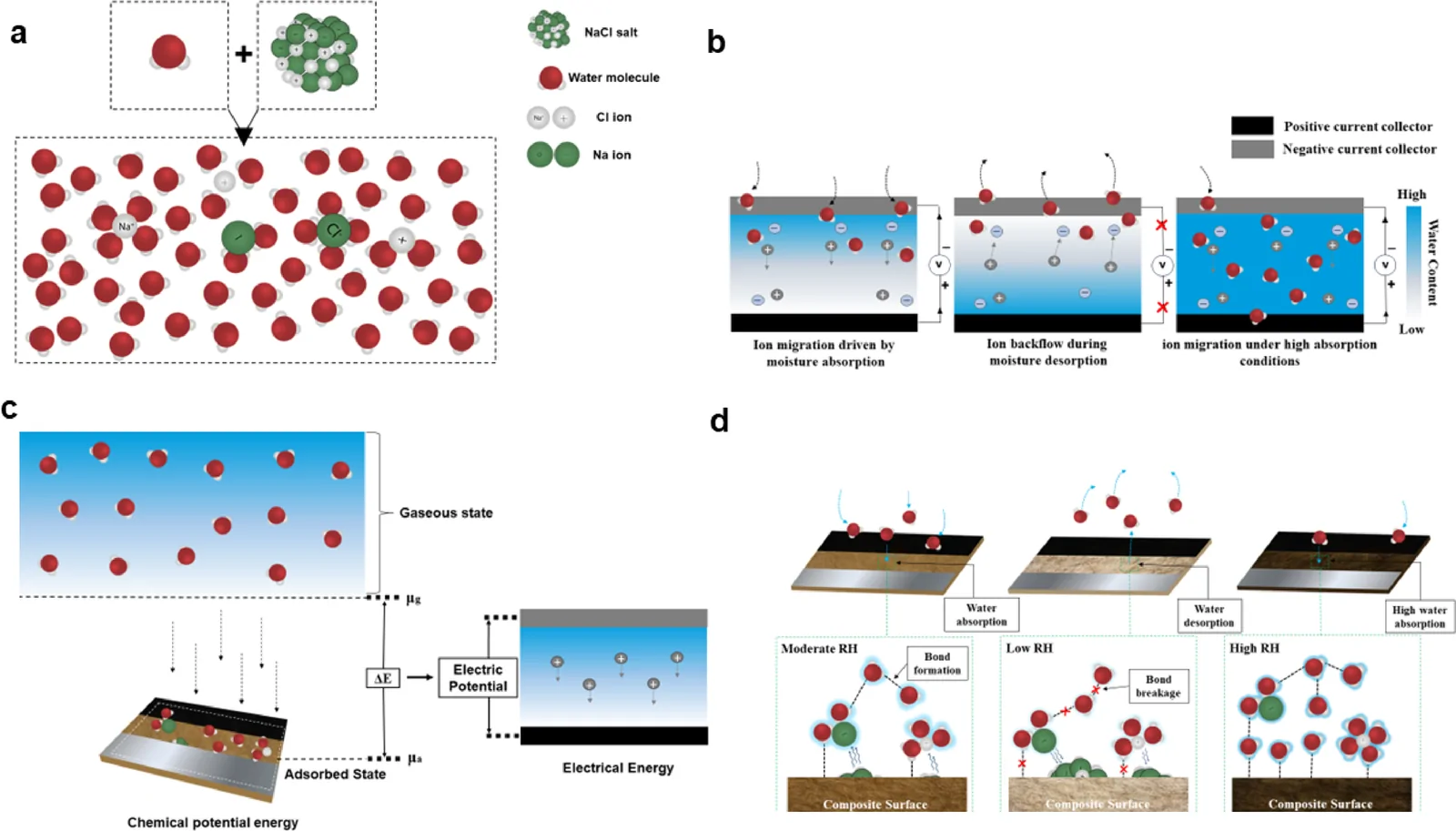
Working mechanism of the MEG driven by moisture interaction. (**a**) Dissolution of crystalline NaCl in absorbed water, forming hydrated Na^+^ and Cl⁻ ions. (**b**) Directional ion migration during moisture absorption under a humidity gradient. (**c**) Conversion of chemical potential gradients into an electrical potential difference between the two electrodes. (**d**) Schematic illustration of ion migration behaviour under low, moderate and high relative humidity (RH) conditions.

The electrical output arises from the formation and maintenance of an internal moisture gradient and an associated ionic concentration gradient across the composite (Supplementary Fig. 13). Upon exposure to relative humidity (RH), the composite absorbs water from the surrounding air. The absorbed water dissolves NaCl and induces its dissociation into Na^+^ and Cl⁻ ions (Fig. 2a), in accordance with the principles of electrolyte dissociation^41^. The resulting ions migrate along concentration gradients established during moisture uptake, leading to interfacial charge separation at the asymmetric electrodes and the development of a potential difference across the external circuit (Fig. 2c and Supplementary Fig. 14). This directional ion migration during moisture absorption under the internal humidity gradient is schematically shown in Fig. 2b.

Water plays dual roles as a medium for ionization and driver of ionic mobility. In hydrophilic matrices, absorbed water forms interconnected transport pathways that facilitate ion motion required for potential formation^42^. In addition, moisture adsorption increases the effective interfacial area and promotes surface charge development, thereby enhancing moisture-induced electrical output. Previous theoretical studies suggest that dissolved salt can increase interfacial charge density in humid environments. In the present system, NaCl provides mobile ions that enhance charge separation during moisture adsorption and enable humidity-driven electricity generation.

The CB embedded in the composite facilitates electric double-layer formation at the electrode interfaces and contributes to interfacial charge separation through counter-ion adsorption^43^. MnO_2_ is known to exhibit pseudocapacitive behavior^44^ and may contribute to interfacial charge storage within the composite; however, quantitative electrochemical characterization (e.g., cyclic voltammetry, electrochemical impedance spectroscopy, and charge–discharge measurements) is required to isolate its specific contribution, which will be addressed in future work. The composite exhibits three RH-dependent regimes (Supplementary Fig. 15). At low RH, the structure remains insufficiently hydrated, and ion mobility is strongly suppressed. At intermediate RH (~ 65%), moisture uptake promotes ionic transport and interfacial charge separation, leading to peak voltage efficiency but moderate power output owing to limited current generation. At high RH, increased water uptake accelerates NaCl dissociation and ion transport, leading to higher current output but also faster voltage relaxation (Fig. 2d and Supplementary Fig. 16). These regimes also appear through surface color variation (Supplementary Fig. 17). According to the Stern model^45^, the interfacial potential is governed by the surface charge density within the electric double layer (EDL). Adsorbed water hydrates the salt phase and enables limited surface protonation/deprotonation and ionic mobility within the composite. Dissolved NaCl provides mobile Na^+^ and Cl⁻ ions, while Na^+^ counter-ion adsorption at CB-rich interfaces can modify the EDL and contribute to interfacial charge separation^46^. The hydrated electrolyte environment in this device is based on NaCl and absorbed atmospheric moisture and is therefore not treated as a strongly acidic or alkaline redox electrolyte. Under such near-neutral conditions, the electrical output is attributed primarily to moisture-gradient-induced Na^+^/Cl⁻ redistribution, interfacial EDL formation, and charge separation at asymmetric electrodes. The persistence of measurable voltage in the CB-only control indicates that a faradaic MnO_2_ reaction is not required for basic voltage generation. MnO_2_ is therefore interpreted as an interfacial modifier that improves voltage retention and charge-buffering behavior, rather than as the sole origin of the moisture-driven electrical output.

The electrical output is governed by the formation and relaxation of internal moisture and ionic gradients rather than by stored chemical energy. Voltage emerges during moisture absorption when these gradients are established, decays as the gradients gradually equilibrate, and recovers only after renewed moisture uptake restores the gradient. This cyclic moisture-driven regeneration distinguishes the system from electrochemical batteries and identifies it as a gradient-driven energy harvester. Electrical signals appear only after moisture adsorption, confirming ambient humidity as the external energy source (Supplementary Fig. 18). Moreover, the MEG enables cyclic electricity generation by repeatedly harvesting energy from environmental moisture through adsorption–desorption cycles.

Moisture-induced structural deformation also contributes to this process. The interaction between water molecules and hygroscopic materials induces structural deformation, commonly observed as swelling (Supplementary Fig. 19) during moisture absorption and shrinking during desorption^47^. At elevated RH, the composite rapidly absorbs water and becomes hydrated, whereas at reduced RH it releases moisture and dehydrates (Supplementary Figs. 20 and 21). The lignocellulosic and cellulose-based components are intrinsically hydrophilic due to polar functional groups such as hydroxyl and carbonyl moieties, which adsorb water via hydrogen bonding^48^ (Supplementary Fig. 22). This dynamic humidity response is essential for sustaining repeated moisture-induced charge generation in the composite.

### Electrical measurements

Open-circuit voltage (V_oc_) and short-circuit current (I_sc_) were measured using a digital source meter (Keithley 2450). Samples were mounted between two metallic electrodes connected to the asymmetric electrode layers and placed inside a sealed environmental chamber with programmable relative humidity (RH) control. Relative humidity was adjusted using saturated salt solutions and monitored using a calibrated digital hygrometer positioned adjacent to the sample. At each RH level, the device was allowed to equilibrate for at least 10 min prior to electrical measurement to ensure stabilization of moisture content and ionic transport pathways. All measurements were performed at 25 ± 1 °C. Open-circuit voltage was recorded under zero-load conditions, while short-circuit current was measured by directly connecting the two electrodes through the source meter in current-measurement mode. Voltage-time and current-time responses were recorded during controlled humidity changes and under external load conditions where indicated. For LED demonstration experiments, the device was connected directly to a commercial low-power red LED without external amplification, and the voltage and current were monitored continuously. Signals below the instrumental detection limit were recorded as zero. Each experiment was repeated at least three times using independently fabricated samples, and representative data are reported in this study.

### Effective two-probe DC conductance/conductivity measurements

The effective two-probe DC conductance/conductivity of the coated composite was evaluated using copper electrodes separated by a fixed distance of 2.0 cm. Rectangular samples with known dimensions were placed between the electrodes under consistent mechanical contact. Because the composite contains mobile ions, absorbed moisture, and interfacial charge-storage sites, the measured electrical response is not interpreted as purely electronic or purely ohmic conductivity. Instead, the values are reported as effective two-probe DC conductivity under identical experimental conditions. Resistance values were obtained after signal stabilization using a digital multimeter (UNI-T UT33D+) under controlled ambient conditions. The effective two-probe DC conductivity (σ_eff_) was estimated from the measured resistance (R) using the relation,

1 $$\:R = \frac{L}{{\sigma _{{eff}} A}}$$

where L is the electrode separation distance and A is the cross-sectional area of the sample. σ_eff represents the effective conductivity. These values were used only for relative comparison between coating densities and composite configurations. Separation of ohmic resistance, ionic transport, interfacial polarization, and capacitive/EDL contributions would require electrochemical impedance spectroscopy (EIS); therefore, no mechanistic claim is made from the conductivity values alone.

## Results and discussion

### Electricity generation of MEG

**Fig. 3:**
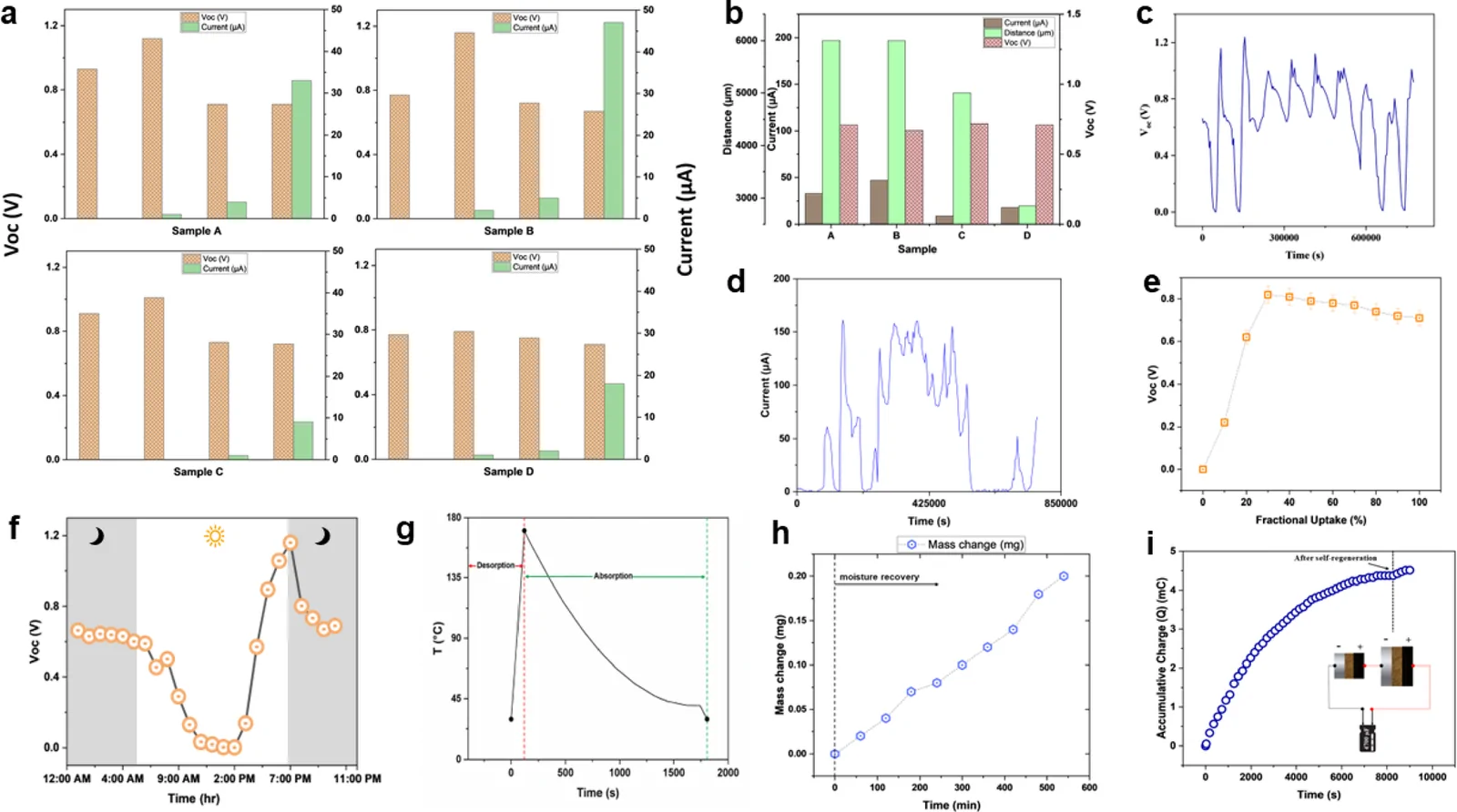
Electricity generation characteristics of the MEG. (**a**) Open-circuit voltage (V_oc_) and short-circuit current (I_sc_) generation of the different sample types. (**b**) Effect of the distance between current collectors on electricity generation. (**c**) Continuous measurement of V_oc_ and (**d**) I_sc_ under open environmental conditions for 216 h (9 days). (**e**) Relationship between V_oc_ and fractional water uptake which shows an asymmetric dependence with an optimum hydration point. (**f**) Voltage variation of a single MEG composite over a 24 h period under ambient conditions. (**g**) Effect of rapid heating that causes water desorption and slower reabsorption during cooling state (**h**) Mass variation of the MEG composite during the moisture recovery state. (**i**) Charge accumulation in a 4,700 µF capacitor connected to a single MEG composite.

Electrical output is generated only when a moisture gradient is established across the composite, consistent with the proposed gradient-driven mechanism (Supplementary Fig. 23). Under humid conditions, the device produced an open-circuit voltage (V_oc_) up to ~ 1.16 V and a short-circuit current (I_sc_) in the microampere range (Fig. 3a). Electrical output was observed only when the two electrodes were placed at opposite sides of the composite; no signal was detected when both electrodes were positioned on the same region, confirming that a spatial moisture gradient is required for charge generation^2^.

The separation distance between the two electrodes strongly influenced the electrical response (Fig. 3b). Variations in V_oc_ and I_sc_ reflect changes in contact resistance and local moisture distribution, indicating that efficient ion transport pathways and asymmetric hydration profiles are essential for maximizing output. Long-term measurements under ambient conditions revealed that both V_oc_ and I_sc_ fluctuate in response to environmental humidity and temperature (Fig. 3c, d). These variations demonstrate that the electrical response is governed by external moisture availability rather than by stored chemical energy. When fully hydrated, the composite continued to exhibit electrical output until moisture gradients gradually equilibrated^30,49^ (Fig. 3e). This behaviour reflects relaxation of the internal humidity gradient rather than sustained electrochemical operation. Accordingly, the output decayed when moisture was lost and recovered only after renewed moisture absorption from the surrounding environment.

To clarify the functional contributions of the individual components, a series of control composites was fabricated by selectively omitting cigarette butt fibers (CA), MnO_2_, or NaCl while preserving the same structural configuration. The fully assembled device exhibited a pronounced humidity-dependent electrical response, whereas all control samples showed substantially reduced or negligible output across the same relative humidity range (Supplementary Fig. 24, 26). The composite prepared without NaCl produced almost no detectable voltage or current, indicating that mobile ions generated by salt dissolution play a critical role in charge transport and potential formation. Removal of MnO_2_ resulted in a marked reduction in open-circuit voltage compared with the full MEG device, indicating that the carbon-based electrode layer alone provides limited interfacial charge separation under identical humidity conditions. (Supplementary Fig. 27, 28). To quantitatively evaluate the contribution of cigarette butt-derived cellulose acetate (CA), control composites were fabricated without the CA fiber network while maintaining identical geometric dimensions, electrode configurations, NaCl loading, temperature, and RH stabilization conditions. In the humidity-dependent control experiment shown in Supplementary Fig. 24, the CA-free control produced only a very small open-circuit voltage across the tested RH range, remaining below approximately 0.06 V, whereas the full MEG device produced substantially higher voltage under the same conditions. At the RH condition selected for direct comparison, the CA-free device showed more than 90% lower Voc than the full MEG device. The same decreasing trend was also observed for Isc and calculated power output/power density, as summarized in Supplementary Table S5 and Supplementary Figs. 25, 26. Therefore, the cellulose acetate fiber network is not claimed to act as a highly hygroscopic active material; rather, it functions as a porous structural scaffold that assists moisture diffusion, salt distribution, and continuity of ionic transport pathways. This supports the conclusion that cellulose acetate contributes structurally to the moisture-driven output by improving the internal distribution of salt and water within the composite matrix. These results support the interpretation that the observed electrical output does not originate from any single component but arises from their synergistic interaction under a sustained humidity gradient. To further evaluate the contribution of MnO_2_, an additional control device was fabricated using a carbon black (CB) coating without MnO_2_ while maintaining identical structural configuration, NaCl loading, electrode arrangement, and humidity conditions. Under identical humidity conditions, the CB-only control device produced a measurable voltage, confirming that moisture-induced ionic transport and electric double-layer formation at the carbon interface can generate electrical output even in the absence of MnO_2_. However, compared with the CB-only device, the MnO_2_-containing MEG showed a higher peak Voc and slower voltage decay, as summarized in Supplementary Table S6 and Supplementary Figs. 27, 28. This indicates that MnO_2_ improves voltage retention and interfacial charge stability under humid conditions. Because CV, EIS, and galvanostatic charge-discharge measurements were not performed, we do not claim that the MnO_2_ contribution has been quantitatively isolated as a reversible faradaic process. Instead, the present control experiment supports a conservative interpretation; the CB network provides the primary EDL/ionic-gradient response, while MnO_2_ improves the effective interfacial charge-buffering behavior of the composite.

Thermal perturbation experiments further supported the moisture-driven nature of the response. Upon illumination, V_oc_ decreased sharply and subsequently recovered when the light source was turned off (Fig. 3f). This behaviour is attributed to photothermal heating of the carbon-containing electrode layer, which accelerates water desorption and reduces the internal moisture gradient. Another analysis shows that under rapid heating water desorption occurs faster than absorption during cooling and leading to asymmetric voltage recovery dynamics (Fig. 3g). Another key observation is that the composite mass, which reflects its moisture content, strongly influences electrical output. When exposed to elevated temperature, rapid water loss occurs and the voltage decreases sharply. Upon re-exposure to ambient conditions, gradual moisture reabsorption takes place, accompanied by recovery of the electrical signal (Fig. 3h). These results demonstrate that device performance is governed by hydration state; output diminishes with moisture loss and reappears only as water uptake restores the internal moisture and ionic gradients. Progressive recovery of V_oc_ during natural adsorption enables repeated humidity-driven charge generation and confirms that electricity production is controlled by environmental moisture rather than by stored chemical energy (Fig. 3i).

**Fig. 4:**
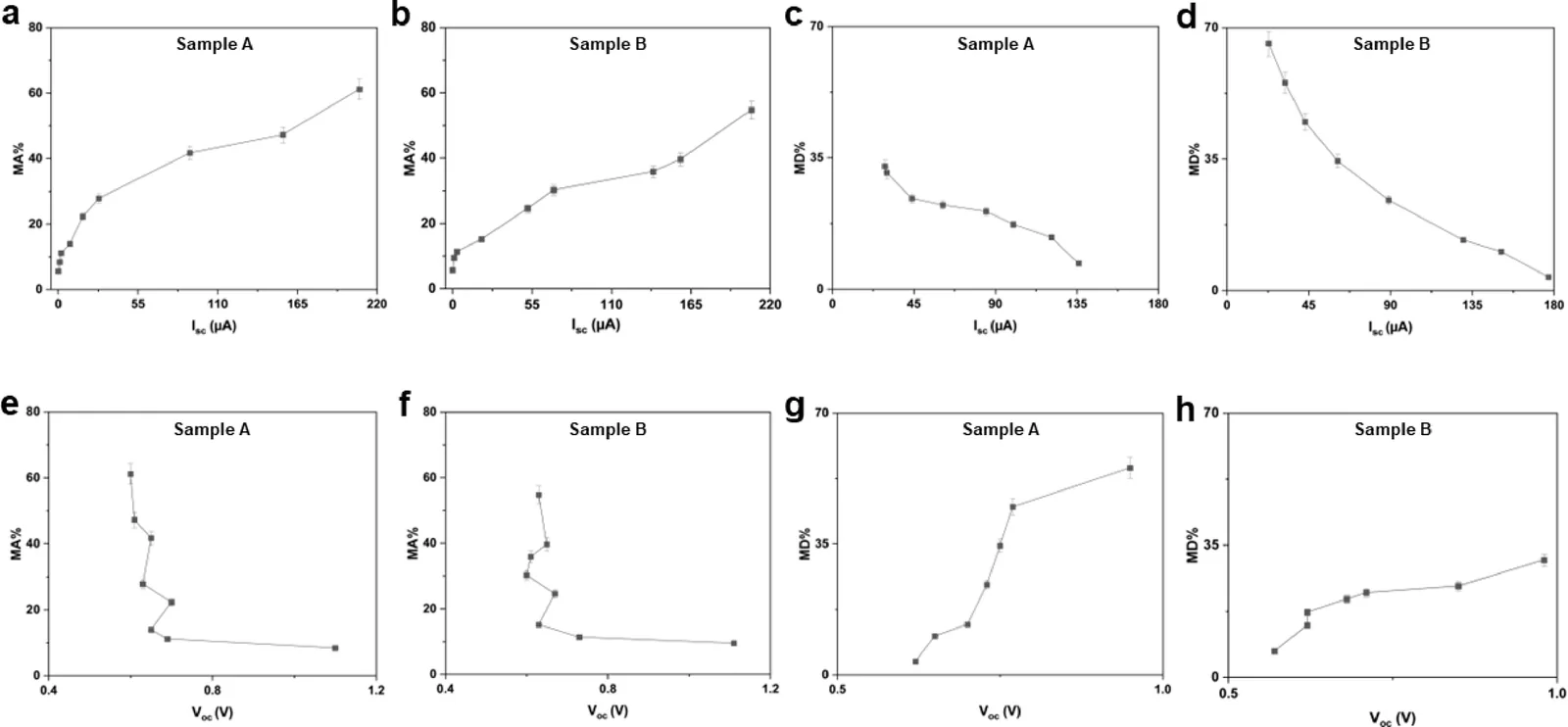
Correlation between moisture and electrical performance of the MEG. (**a**) Relationship between current (I_sc_) generation and moisture absorption (MA%) for Sample (A) and (**b**) Sample (B) (**c**) Decrease in I_sc_ with moisture desorption (MD%) for Sample A and (**d**) Sample B. (**e**) Asymmetric dependence of open-circuit voltage (V_oc_) on MA% for Sample A and (**f**) Sample B. (**g**) Correlation between V_oc_ and MD% for Sample A and (**h**) Sample B, demonstrating the strong coupling between hygroscopic behavior and electrical response.

To further understand the dynamic behaviour of the composite, time-resolved moisture absorption and desorption experiments were conducted under controlled environmental conditions on two representative samples (Samples A and B). During moisture absorption (MA%), the short-circuit current (I_sc_) increased proportionally (Fig. 4a, b), whereas moisture desorption led to a corresponding decline in current (Fig. 4c, d). In contrast, the open-circuit voltage (V_oc_) exhibited a nonlinear dependence on both MA% (Fig. 4e, f) and MD% (Fig. 4g, h), forming an asymmetric bell-shaped profile. This behaviour defines an optimal hydration window in which ion mobility and interfacial charge separation are maximized.

Excess moisture caused partial screening of interfacial charge and a reduction in V_oc_, consistent with dielectric dilution effects. The faster increase in I_sc_ during moisture uptake reflects rapid activation of mobile ions, whereas the slower decay during desorption is attributed to the retention of bound water and delayed ionic deactivation within the porous matrix. Time-resolved measurements further show that current rises sharply upon initial moisture exposure and decays gradually during drying (Supplementary Fig. 29), while V_oc_ evolves more slowly and fluctuates as the system approaches moisture equilibrium (Supplementary Fig. 30).

### Geometry optimization for MEG output

**Fig. 5:**
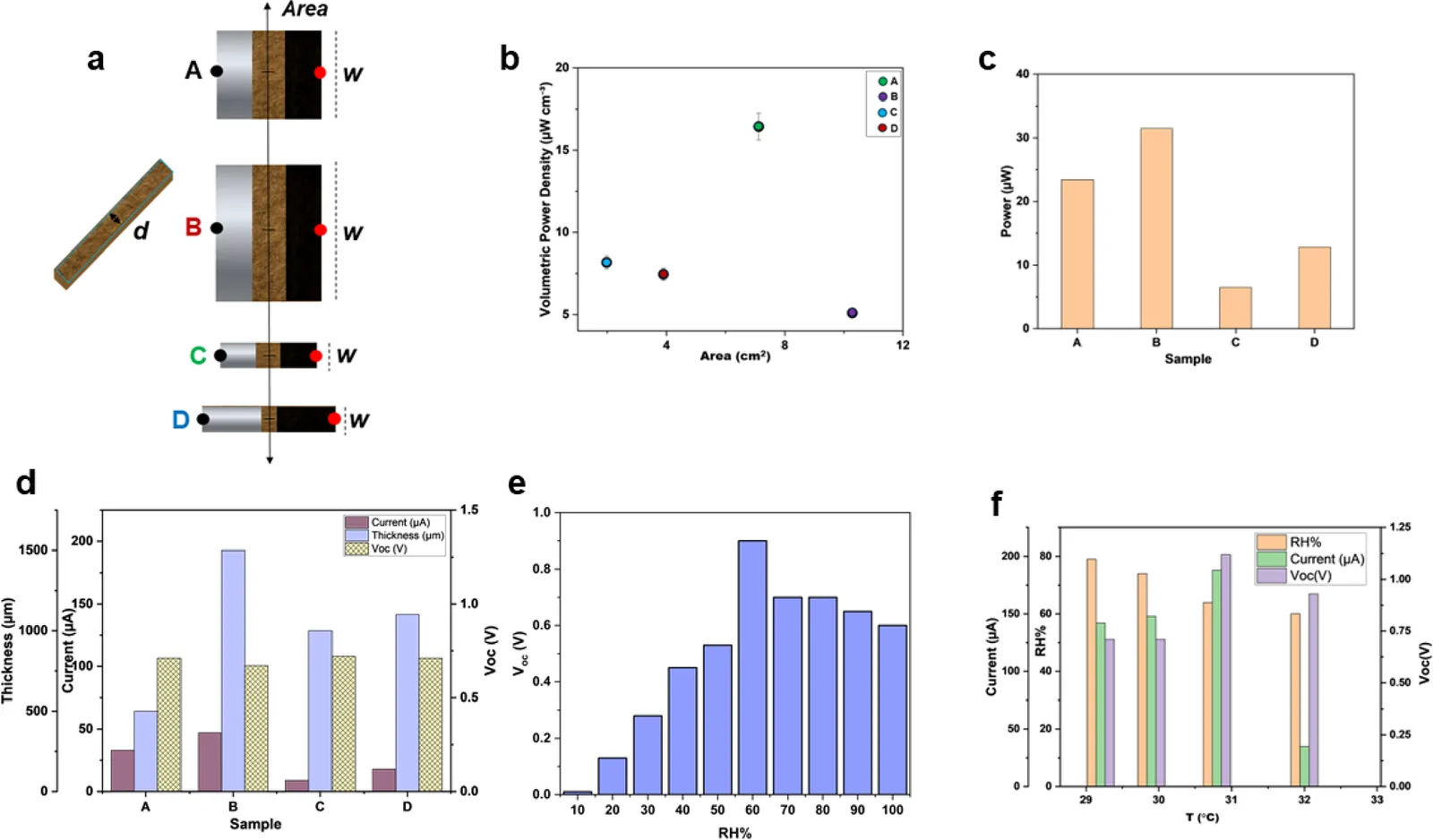
Geometry optimization for enhanced MEG output performance. (**a**) Illustration of four MEG configurations (Samples A, B, C and D) with different length, width, and surface area. (**b**) Volumetric power density of each sample plotted as a function of geometric area. (**c**) Power output of MEGs with different sizes. (**d**) Influence of composite thickness on open-circuit voltage (V_oc_) and short-circuit current (I_sc_). (**e**) Electrical performance of the MEG under varying relative humidity (RH) conditions. (**f**) Effect of environmental factors, including humidity and temperature, on electricity generation efficiency.

The preliminary observation in MEG was that a larger area exhibited an increase in output by enhancing I_sc_^2^. However, further investigation showed that final power output depends more on ion transport efficiency and moisture distribution rather than total surface area. To validate this, four MEG samples with different surface areas but identical length, width, and thickness were fabricated (Fig. 5a). Power Density was estimated as

2 $$\:P=\frac{{V}_{oc}.{I}_{sc}}{4(A.d)}$$

where *A* is the projected area and *d* is thickness. Sample A showed the highest volumetric power density of approximately 16.44 µW cm^−3^ (Fig. 5b). Based on its measured density of approximately 2.41 g cm^−3^, this corresponds to a gravimetric power density of approximately 6.82 µW g^−1^. Sample B showed the highest gravimetric power density of approximately 15.74 µW g^−1^ (Supplementary Fig. 31) and confirmed the highest total power output among all tested samples (Fig. 5c). It showed a value approximately five times greater than that of the lowest-powered sample. (Supplementary Fig. 32) However, its volumetric power density was lower (≈ 5.1 µW cm^−3^). This contrast highlights the importance of balancing material efficiency per unit mass and total power output per unit volume in composite. The optimal composite thickness increases voltage and current and produces superior power (Fig. 5d). The structure is consistent with reduced internal resistance and improved ion transport, which gives higher performance. Thus, careful geometric balancing is essential for improving MEG performance.

The electrical response of the MEG is strictly governed by ambient relative humidity (RH). V_oc_ increased with RH and reached a maximum near ~ 65%, followed by a gradual decline at higher RH (Fig. 5e). This behaviour reflects the competing roles of enhanced ion dissociation and dielectric screening at elevated moisture levels. The presence of NaCl, with a deliquescent RH near 75% at 25 °C, facilitates moisture uptake and provides mobile ions that support charge separation under humid conditions.

In contrast to previously reported MEG systems, which typically required highly specific environmental conditions and exhibited only transient electrical responses^8,50^. The MEG demonstrated measurable electrical performance across a wide range of temperatures and relative humidity levels (Fig. 5f). This response arises from the combined effects of hygroscopic salts and porous composite structure, which help retain moisture and support measurable humidity-dependent voltage under varying environmental temperature^51^ However, excessive heating accelerates moisture loss and suppresses ion mobility, leading to reduced voltage and current (Supplementary Fig. 33). Moderate ambient temperatures promote moisture retention and enhance electrical response (Supplementary Fig. 34). Collectively, these results demonstrate that the device operates as a humidity-driven energy harvester in which electrical output arises from the formation and relaxation of moisture and ionic gradients, rather than from stored chemical energy.

### Functional integration and application prospects of the MEG

**Fig. 6:**
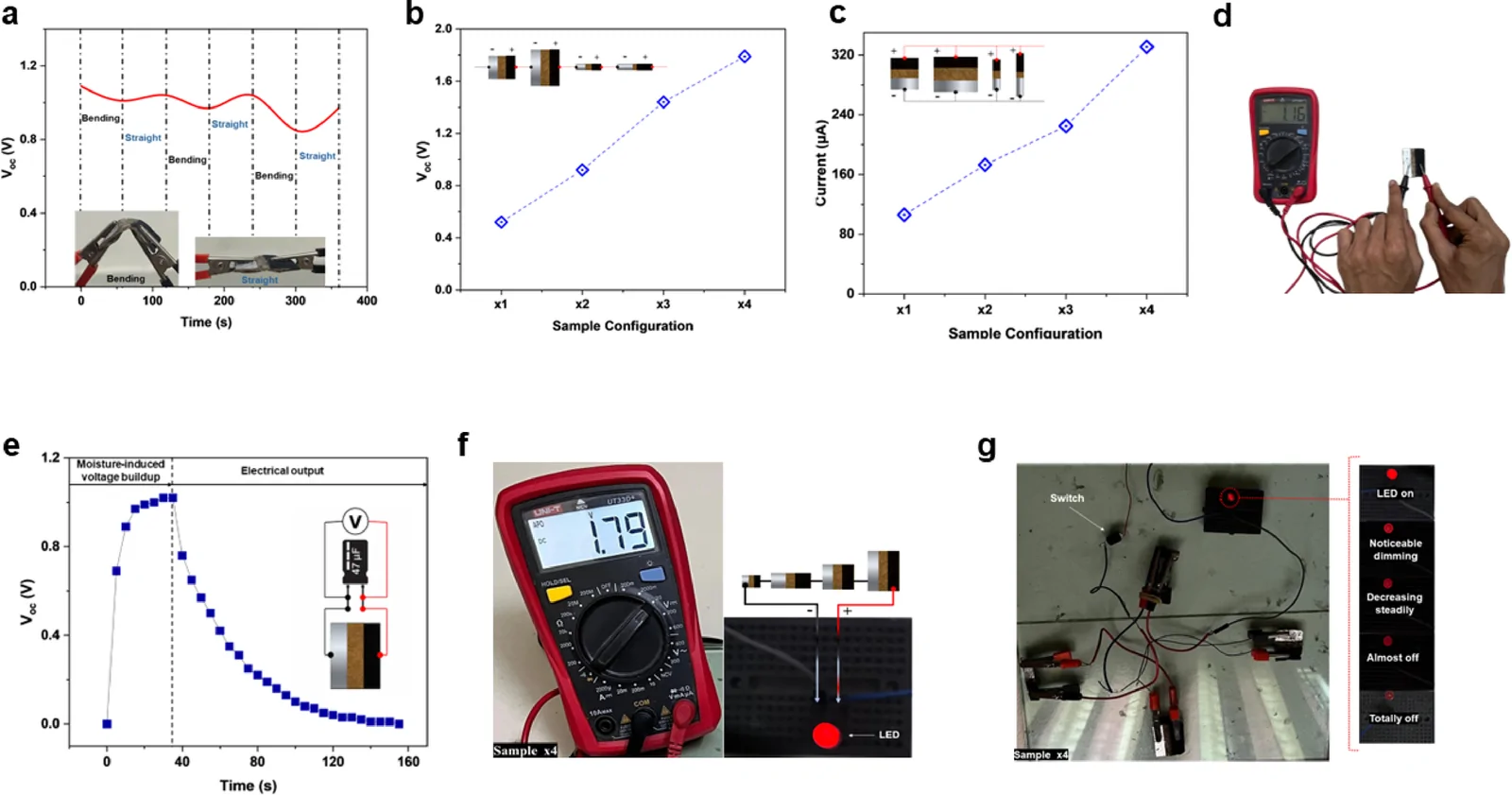
Performance and integration of MEG units for energy harvesting and device operation. (**a**) V_oc_ stability under mechanical deformation. (**b**) V_oc_ output versus the number of serially connected MEG units. The inset is serial circuit. (**c**) I_sc_ output for parallel configurations. The inset is parallel circuit. (**d**) Highest V_oc_ recorded from a single MEG. (**e**) Charge accumulation in a 4,700 µF capacitor powered by a single MEG under humid conditions. (**f**) Direct powering of a red LED using four MEG units connected in series. (**g**) LED illumination over three hours demonstrates direct low-power operation under favorable humid conditions while a moisture gradient is maintained.

The MEG exhibits strong potential as a humidity-induced energy-harvesting platform for low-power applications. Its fabrication is cost-effective, as the entire system is constructed from waste-derived materials (Supplementary Fig. 35), enabling upcycling while promoting sustainability and resource efficiency. The composite structure demonstrates excellent mechanical flexibility and maintains measurable electrical output under bending deformation (Fig. 6a), supporting its suitability for wearable and deformable devices. Scalable electrical output was achieved by connecting multiple MEG units in series and parallel configurations. Series connection increases the open-circuit voltage (V_oc_) (Fig. 6b), whereas parallel connection enhances the short-circuit current (I_sc_) (Fig. 6c) consistent with additive moisture-induced potential generation. The highest V_oc_ obtained from a single MEG was 1.16 V at 65% RH (Fig. 6d). To place the performance of the present moisture-driven electric generator (MEG) in context, its electrical output was compared with previously reported moisture-driven energy harvesting systems operating under comparable relative humidity conditions. Supplementary Table 4 summarizes representative systems in terms of material composition, operating humidity range, open-circuit voltage, and reported power density. The present MEG delivers an open-circuit voltage of 1.16 V and a volumetric power density of 16.44 µW cm^−3^, which is comparable with several reported MEG systems based on hygroscopic and ionically conductive materials under similar humidity conditions. This comparison provides a qualitative benchmarking rather than a strict quantitative equivalence due to differences in device geometry, measurement configurations, and reporting conventions across literature. In the series configuration, voltage enhancement arises from the cumulative ionic potential differences established across individual units under identical humidity gradients. For practical operation, the electrical output of the MEG can be coupled directly to external electronic components. The generated electricity can be temporarily buffered using a capacitor to provide regulated output when required (Fig. 6e), or the device can be directly connected to low-power loads under humid conditions (Fig. 6f). When four MEG units were connected in series and exposed to 80% RH at 30.5 °C, the combined system produced 1.79 V and 323 µA (Supplementary Fig. 36), which was sufficient to illuminate a 3 mm red LED for over 180 min or 3 h. However, after 3 h of operation, the MEG system displayed a measurable voltage, although no current was observed (Supplementary Fig. 37). The time-dependent voltage and current delivered to the LED during operation are shown in Supplementary Fig. 38 and 39, confirming measurable electrical output under maintained humid conditions (Supplementary Movie 1). Illumination persisted only while a moisture gradient was maintained, confirming ambient humidity as the driving energy source.

The strong dependence of output on relative humidity highlights the suitability of the MEG for passive, humidity-responsive power generation and long-duration micro-power harvesting. Unlike most previously reported moisture-induced generators that require capacitors^52^ as intermediate buffers, the MEG was capable of directly driving an LED under favorable humidity conditions (Fig. 6g). By weight, the MEG delivers 1.52 V at 1.48 g and provides a strong balance between voltage and mass. In contrast conventional 1.5 V batteries like Olympic, Sunlight, Murata LR44^53^, and Enfuel Soft Battery^54^ are significantly heavier. It offers a higher voltage-to-weight ratio making it well suited for lightweight wearable applications (Supplementary Table 1). This comparison is provided only as a mass-normalized voltage reference and does not imply that the MEG functions as an electrochemical battery or energy-storage device.

### Baqui’s moist electric generation model (Baqui’s MEG model)

The present MEG system combines hydrophilic materials and hygroscopic salts to facilitate moisture absorption, ion dissociation, and subsequent charge transport under humid conditions. These coupled processes form the operational basis of the present moisture-driven electric generator. To provide a conceptual interpretation of the observed relationship between moisture absorption and electrical output, this study proposes a preliminary phenomenological framework referred to as Baqui’s Moist Electric Generation Model (Baqui’s MEG Model).

The proposed conceptual model suggests that:

*“In a system combining hydrophilic materials, hygroscopic salts, and atmospheric moisture, increased moisture absorption promotes ion dissociation and ionic transport, which can generate measurable electrical output under maintained humidity-gradient conditions when connected to conductive current collectors.”*

This model describes the observed relationship between moisture uptake and electrical generation in the present MEG system. Hydrophilic materials facilitate atmospheric moisture adsorption, while hygroscopic salts promote the formation of mobile ions within the hydrated composite matrix. As moisture uptake increases, ion availability and ionic mobility increase, supporting charge separation and directional ion transport. When asymmetric conductive current collectors are present, these coupled processes produce a measurable electrical potential difference under maintained humidity-gradient conditions.

### Conceptual mathematical framework for Baqui’s MEG model

To provide a conceptual mathematical framework for interpreting the present experimental observations, we propose a simplified phenomenological description based on established transport principles, including Fick’s Law of Diffusion^55^, the Nernst-Planck equation^56^, the Nernst equation^57^, and Ohm’s Law^58^.

In the present MEG system, moisture absorption by hydrophilic materials facilitates ion dissociation from hygroscopic salts and promotes ionic transport under a maintained humidity gradient. The flux of ions (*J*) is governed by Fick’s First Model of Diffusion^55^, expressed as:

3 $$\:J=-D\frac{dc}{dx}$$

where *J* represents the ionic flux, *D* is the diffusion coefficient of ions, $\:\frac{dC}{dx}$ denotes the concentration gradient of mobile ions across the material. The equation describes ion migration from high to low concentration regions driven by moisture-induced dissociation that leads to charge separation and electricity generation. As moisture absorption (*A*) increases, the ion concentration (*C*) within the system exhibits a proportional relationship, expressed as:

4 $$\:C={k}_{A}A$$

where *k*_*A*_ is a constant dependent on material hygroscopicity and salt dissociation efficiency. This relationship highlights that greater moisture uptake increases free ion availability which enhances ionic conductivity and supports charge transport. The rate of ion generation follows:

5 $$\:\frac{dc}{dt}=\alpha\:A$$

Here $\:\frac{dC}{dt}$ represents the rate of change of ion concentration over time and *α* is a material-dependent coefficient that characterizes the efficiency of ion dissociation per unit of absorbed moisture. The equation illustrates that higher moisture absorption leads to a proportional increase in ion generation. This transport behavior is governed by the Nernst-Planck equation, expressed as:

6 $$\:J=-D\frac{dc}{dx}+\mu\:CE$$

where *D* is the ion diffusion coefficient, denotes the concentration gradient, *µ* is the ion mobility, *C* is the ion concentration, and *E* represents the applied electric field. The first term in Eq. 6 describes ion diffusion driven by concentration gradients as per Fick’s Law. The second term captures ion migration under an electric field which enhances directed charge transport. Furthermore, the generated voltage (*V*) is dictated by the ion concentration gradient across the system, as described by the Nernst equation:

7 $$\:V=\frac{RT}{zF}\mathrm{l}\mathrm{n}\left(\frac{{C}_{out}}{{C}_{in}}\right)$$

where *R* is the universal gas constant, *T* is the absolute temperature, *z* is the charge number of the ions, F is the Faraday constant, *C*_*out*_ and *C*_*in*_ are the ion concentrations at the two points (electrodes/current collectors). As moisture absorption increases, the higher ion concentration gradient strengthens the electrochemical potential and enhances the voltage output. The relationship between V and *A* can be expressed as:

8 $$\:V\propto\:\mathrm{l}\mathrm{n}\left(A\right)$$

Here *k*_*V*_ is a constant that includes material-specific factors. The equation indicates that *V* increases logarithmically with *A* as higher ion concentration enhances electrochemical potential. Besides, *I* generated is related to *V* and *R* (electrical resistance) of the material, based on Ohm’s law which expressed as:

9 $$\:I=\frac{V}{R}$$

According to this relationship, for a given material, an increase in *V* will lead to an increase in the *I* generated, provided that the *R* remains constant. The system resistance *R* depends on ionic conductivity, geometry and ion transport efficiency. Increased *A* enhances conductivity which lowers resistance and boosts current output. The *R* is given by:

1 $$\:R=\frac{L}{\sigma\:A}$$

where *L* represents the length of the conductive path through the material, *σ* is the ionic conductivity, and *A* is the cross-sectional area through which the ions are transported. This relationship shows *R* decreases with higher ionic conductivity or cross-sectional area and increases with material length. As *A* increases ionic conductivity improves which reduces *R* and increases, *I* as described by Ohm’s law (Eq. 9). Again, as *A* increases, the *σ* is directly proportional to *A*, which can be expressed as:

10 $$\:\sigma\:\propto\:A$$

Thus, the generated current *I* can be written as:

11 $$\:I=\frac{kVln\left(A\right).k\sigma\:A}{L}$$

where, *k* is a proportionality constant. Here, *k*_*σ*_ reflects material properties including ion dissociation efficiency. The power output (*P*) of the system can be expressed as,

12 * P=VI*

Substituting the *V* and *I* from Eqs. (8) and (11), respectively, the power output becomes:

13 $$\:P=\left(kVln\left(A\right)\right)\frac{kVk\sigma\:Aln\left(A\right)}{L}$$

Simplifying, we get:

14 $$\:P=\frac{{k}^{2}VK\sigma\:A{ln}^{2}\left(A\right)}{L}$$

Finally, the derived model shows that the *P* is directly proportional to *A* with a logarithmic dependence, expressed as:

15 $$\:P\propto\:A{ln}^{2}\left(A\right)$$

This equation supports Baqui’s MEG Model by showing that increased moisture absorption increases ion concentration which enhances ionic conductivity and improves power output highlighting the key role of moisture in system performance. This behavior aligns with the fundamental principles of ion transport and electrochemical potential and shows the importance of optimizing moisture absorption to maximize power output.

### Validation and applicability of Baqui’s MEG model

To evaluate the proposed model that describes the relationship between moisture absorption and power generation, controlled experiments were conducted using MEG. The mathematical model proposes that the generated power output, P depends on the moisture absorption capacity, A by following the relationship:

16 $$\:P=KA{ln}^{2}\left(A\right)$$

This study experimentally evaluated this equation and found an *R²* value of 0.95. (Supplementary Table 2). This high coefficient indicates good agreement between the model and the present experimental dataset. The experimental data and the theoretical curve align well across the entire moisture absorption range (Supplementary Fig. 40). The *K* value of 0.155 is a material-specific constant reflecting ion mobility, porosity, interfacial conductivity and internal moisture-driven potential gradients. The key consideration is that Baqui’s MEG model establishes a simplified theoretical framework for interpreting the present MEG system and related humidity-driven devices for understanding moisture-electric generation. Beyond our system, we qualitatively aligned the model with trends from previous MEG devices. While the equation links moisture absorption to power output, earlier studies often correlate power output with relative humidity (RH%) or RH and voltage/current. (Supplementary Fig. 41). However, as RH rises, moisture absorption by materials increases proportionally^59^ due to the greater uptake by hydrophilic and hygroscopic materials, particularly at high RH. Our experimental data (Supplementary Table 3) further suggest that RH may serve as an approximate indicator of moisture absorption under comparable conditions. However, MEG systems vary in material properties, fabrication methods, structure and measurement conditions. Despite these differences, our equation aligns with trends in previous MEG studies showing that power output rises with absorbed moisture of its natural logarithm. This suggests that moisture influences electricity generation through a nonlinear trend. These results suggest Baqui’s MEG Model provides a preliminary conceptual framework for understanding moisture-induced electricity generation for interpreting related humidity-responsive MEG systems.

## Conclusion

In summary, we have developed a moisture-driven electric generator (MEG) by engineering a multifunctional composite structure entirely from waste-derived materials. The device produces reproducible electrical output governed by moisture-induced ionic transport under ambient humid conditions. Integration of wild sugarcane fibers and recycled cigarette butt fibers with a salt-based electrolyte derived from e-waste enables the fabrication of a flexible and scalable humidity-responsive energy-harvesting platform. Electrical output originates from the formation of internal moisture and ionic concentration gradients across asymmetric electrodes rather than from stored chemical energy. A single MEG achieved an open-circuit voltage of up to 1.16 V and a volumetric power density of 16.44 µW cm^−3^. Electrical output can be increased by connecting multiple MEG units in series or parallel, providing a scalable strategy for long-duration, low-power energy harvesting. Comparison with previously reported moisture-driven generators demonstrates that the present MEG exhibits competitive electrical performance under practical environmental conditions (Supplementary Table 4). We also present ‘Baqui’s MEG Model’ as a preliminary conceptual framework linking moisture absorption, ion dissociation, and electricity generation in the present MEG system. These findings highlight both practical viability and deeper insights into moisture–material interactions for next-generation energy systems.

## Supplementary Information

Below is the link to the electronic supplementary material.

## Data Availability

All data supporting the findings of this study are available within the Source Data file and the Supplementary Information associated with this article.
