# Supplementary material for: Sustainable moisture-driven electricity generation using waste materials
Source: Sci Rep. 2026 Jul 14;16:27522. doi: 10.1038/s41598-026-61180-8 (PMC13538561; doi:10.1038/s41598-026-61180-8)
Supplement: Supplementary file 2 [file 41598_2026_61180_MOESM2_ESM.docx]

**Supplementary Information**

**Moisture-driven Composite Battery from Waste Materials for Sustainable Energy Harvesting.**

Aman Ul Azam Khan^1^, Nazmunnahar Nazmunnahar^2^*, Mortuza Hasan^1^, Abdul Baqui^3^

^1^Department of Textile Engineering, BGMEA University of Fashion & Technology, Dhaka 1230, Bangladesh.

^2^Department of Earth and Environment Science, The University of Manchester, M13 9PL, United Kingdom.

^3^School of Engineering and Technology, Central Queensland University (CQU), Melbourne, VIC, Australia.

*Corresponding author: Nazmunnahar Nazmunnahar (nazmunnahar@manchester.ac.uk)

**Supplementary Fig. 1** Collection of Wild Sugarcane Stalks as Naturally Dispersed Biomass Waste.

**Supplementary Fig. 2** Extraction of Fibers from Discarded Cigarette Butts.

**Supplementary Fig. 3** Spraying of Boiled Saline Solution onto Wild Sugarcane Fiber Sheet

 **Supplementary Fig. 4** Fiber Structured in a Square Grid of Horizontal and Vertical Lines

**Supplementary Fig. 5** Moisture Absorption Behavior of Hygroscopic Sea Salt (NaCl)

**Supplementary Fig. 6** Comparison of Salts with Distinct Deliquescence Relative Humidity (DRH) Levels.

**Supplementary Fig. 7** Time-dependent water uptake of sea salt in bulk and as thin layers on glass and composite substrates. Bulk salt (red) absorbs more slowly than the composite-supported layer (blue) initially, but surpasses the glass-supported layer (black) during 4 h to 9 h. The composite shows the fastest uptake in the first ~8 h, while the glass-supported layer overtakes both at longer times (>11 h). Water uptake continues to rise throughout the 15 h test, showing that equilibrium was not yet reached.

**Supplementary Fig. 8** Insolubility of CB–MnO₂ Mixture in Saline Solution (Sea Salt + Water).

**Supplementary Fig. 9** Surface evolution of the composite during CB–MnO₂/salt electrolyte coating, where successive brush–dry cycles lead to progressive black coloration and a final material loading of ~9.5 g cm⁻² after three cycles.

**Supplementary Fig. 10** Conductivity Dependence on Coating Solution Loading Density.

**Supplementary Fig. 11** (a) Moisture absorption (MA%) and (b) moisture desorption (MD%) of the uncoated composite only (CO) and carbon-coated composite (CCC) under varying relative humidity (RH%). The CCC shows higher moisture uptake and desorption which confirms enhanced hygroscopic behaviour critical for efficient moisture-driven power generation in MEG systems.

**Supplementary Fig. 12** (a) SEM characterization of the MECB composite and (b) EDX mapping of NaCl^1^ (1).

**Supplementary Fig. 13** Moisture-Induced Ionic Transport on the Composite Surface

**Supplementary Fig. 14** Ion Concentration Gradient–Driven Potential Generation Between Current Collectors

**Supplementary Fig. 15** Moisture-Driven State Transitions in Composite Structures

**Supplementary Fig. 16** Schematic illustration of the working principle of the MECB

**Supplementary Fig. 17** Surface Color Variations of the Composite Corresponding to Three RH-Dependent States: low RH, moderate RH, and high RH.

**Supplementary Fig. 18** Schematic representation of moisture-driven power generation under natural humidity cycles.

**Supplementary Fig. 19** Moisture-Induced Swelling of Hygroscopic Materials via Water–Material Interactions.

**Supplementary Fig. 20** Humidity-Responsive Hydration and Dehydration Dynamics of the Composite.

**Supplementary Fig. 21** Humidity-Driven Hydration–Dehydration Response of the Fully Coated Composite.

**Supplementary Fig. 22** (a) Water molecules generate hydronium ions, (b) form hydrogen bonds with polar groups, and (c) bind to the composite surface.

**Supplementary Fig. 23** Voltage response of the MECB showing an initial rise followed by exponential decay and stabilization over time.

**Supplementary Fig. 24** Time-resolved current response: (a) sharp rise during moisture absorption and (b) gradual decay during desorption.

**Supplementary Fig. 25** Time-resolved open-circuit voltage (Voc) response: (a) gradual increase during moisture absorption and (b) decrease during desorption.

**Supplementary Fig. 26** Gravimetric power density of samples expressed as power-to-mass ratio.

**Supplementary Fig. 27** Optimized geometry of the MECB to maximize power density. Four configurations were devised, with their power outputs denoted as P0 (Sample C), P1 (Sample D), P2 (Sample B), and P3 (Sample A), respectively.

**Supplementary Fig. 28** MECB maintains performance over a wide temperature range, but extreme heat reduces moisture and lowers voltage output.

**Supplementary Fig. 29** The open-circuit voltage of MECB correlates with moisture absorption and relative humidity.

**Supplementary Fig. 30** Cost analysis of MECB on a per-square-meter basis.

**Supplementary Fig. 31** Connected multiple MECB units in series and parallel increases Voc and Isc.

**Supplementary Fig. 32** Voc and Isc of MECB were measured during LED operation and after the LED was turned off.

**Supplementary Fig. 33** Analysis of experimental data alignment with the theoretical curve across the full range of moisture absorption.

**Supplementary Fig. 34** Relationship between electrical output and relative humidity reported in previous studies^1-5^. (1-5)

**2. Supporting Table**

| **Sample** | **Output Voltage (Voc)** | **Weight (g)** | **Voltage-to-weight  ratio (V/g)** |
| --- | --- | --- | --- |
| MECB (x1) | 0.9 | 0.76 | 1.18 |
| MECB (x2) | 1.18 | 1.19 | 0.99 |
| MECB (x3) | 1.52 | 1.48 | 1.03 |
| Olympic Battery | 1.5 | 15.55 | 0.10 |
| Sunlight Battery | 1.5 | 17.07 | 0.09 |
| Murata LR44 | 1.5 | 2.0 | 0.75 |
| Enfuel Soft Battery | 1.5 | 2.4 | 0.63 |

**Supplementary Table 1** Comparison of voltage, weight, and voltage-to-weight ratio between MECB and conventional 1.5 V batteries.

| **Parameter** | **Value** |
| --- | --- |
| Equation | y = a + b*x |
| Plot | Power (μW) |
| Weight | No Weighting |
| Intercept | -27.93934 ± 7.07943 |
| Slope | 2.92215 ± 0.23989 |
| Residual Sum of Squares | 863.23156 |
| Pearson's r | 0.97722 |
| R-Square (COD) | 0.95495 |
| Adj. R-Square | 0.94851 |

**Supplementary Table 2** Linear regression analysis of MECB

| **Relative Humidity (RH%)** | **Moisture Absorption (MA%)** | **Power (µW)** |
| --- | --- | --- |
| 60 | 5.66 | 0 |
| 65 | 9.43 | 0.82 |
| 70 | 11.32 | 1.54 |
| 75 | 13.20 | 2.52 |
| 80 | 16.98 | 30.40 |
| 85 | 32.08 | 64.35 |
| 90 | 45.28 | 95.55 |
| 95 | 52.83 | 132.60 |

**Supplementary Table 3** Influence of relative humidity on moisture absorption and power output of the MECB

| **Hygroscopic Materials** | **Power Density** | **Open Circuit Voltage** | **Reference** |
| --- | --- | --- | --- |
| Heterogeneous Gels and Carbon Nanotubes | 9.12 μW cm^–2^ | 1.03 V | Huang, Z. et al ^6^ |
| Acrylamide (AAm) and 2-acrylamide-2-methylpropanesulfonic acid (AMPS) | - | 0.89 V | Cheng, Y. et al.^7^ |
| Biological Nanofibrils | 6.3 x 10^-4^ μW cm^−2^ | 0.11 V | Li, M. et al.^8^ |
| Paper | 2.5 x 10^-3^ μW cm^-2^ | 0.25 V | Gao, X. et al.^9^ |
| GO/PAAS Composite | 7.0 x 10^-5^ μW cm^-2^ | 0.6 V | Xue, J. et al.^10^ |
| 3D PPy Framework | 6.9 x 10^-4^ μW cm^-2^ | 0.06 V | Huang, Y. et al.^11^ |
| Hygroscopic ionic hydrogel and carbon black-coated cotton knitted fabric | 0.7 μW cm^–2^ | 0.3 V | Wen, X. et al. ^12^ |
| SSG film (SA–SiO_2_–rGO) | 8.4 x10^-2^ μW cm^–2^ | 0.6 V | Wang, H. et al.^13^ |
| PSSA/PDDA Hybrid Film | 9 x10^-4^ μW cm^-3^ | 1.38 V | Huang, Z. et al.^14^ |
| P(MEDSAH-co-AA) | 1.12 μW cm^-2^ | 0.4 V | Long, Y. et al.^15^ |
| PAN/PSSA Nanofiber Fabrics | 1.48 x10^-2^ μW cm^-2^ | 1.1 V | Sun, Z. et al.^16^ |
| TiO2 Nanowire Networks | 4 μW cm^-2^ | 0.5 V | Shen, D. et al.^4^ |
| PSS/PVA Film | 7.9 μW cm^-2^ | 0.12 V | Yang, W. et al.^3^ |
| Ionic Liquid Film | 12.11 μW cm^-2^ | 0.31 V | Zheng, S. et al.^17^ |
| **MECB (Our Work)** | **16.44 μW cm^-3^** | **1.16 V** | - |

**Supplementary Table 4** Comparative performance of the MECB and previously reported moisture-electric generators (MEGs).

**References**

1	Zhang, Y. *et al.* An asymmetric hygroscopic structure for moisture‐driven hygro‐ionic electricity generation and storage. *Advanced Materials* **34**, 2201228 (2022).

2	Zhao, F. *et al.* Highly efficient moisture-enabled electricity generation from graphene oxide frameworks. *Energy & Environmental Science* **9**, 912-916 (2016).

3	Yang, W. *et al.* Asymmetric ionic aerogel of biologic nanofibrils for harvesting electricity from moisture. *Nano Energy* **71**, 104610 (2020).

4	Shen, D. *et al.* Self‐powered wearable electronics based on moisture enabled electricity generation. *Advanced Materials* **30**, 1705925 (2018).

5	Yan, H. *et al.* Unlocking the potential of hydrogel-electrode electrical double layer for high-performance moisture-enabled electric generators. *Device* **3** (2025).

6	Huang, Z. *et al.* Flexible Moisture-Driven Electricity Generators Based on Heterogeneous Gels and Carbon Nanotubes. *ACS Applied Materials & Interfaces* **17**, 7916-7928 (2025).

7	Cheng, Y. *et al.* Hydrogel‐Based Moisture Electric Generator with High Output Performance Induced by Proton Hopping. *Advanced Functional Materials*, 2500186 (2025).

8	Li, M. *et al.* Biological nanofibrous generator for electricity harvest from moist air flow. *Advanced Functional Materials* **29**, 1901798 (2019).

9	Gao, X. *et al.* Electric power generation using paper materials. *Journal of Materials Chemistry A* **7**, 20574-20578 (2019).

10	Xue, J. *et al.* Vapor‐activated power generation on conductive polymer. *Advanced Functional Materials* **26**, 8784-8792 (2016).

11	Huang, Y. *et al.* All-region-applicable, continuous power supply of graphene oxide composite. *Energy & Environmental Science* **12**, 1848-1856 (2019).

12	Wen, X. *et al.* High‐performance fully stretchable moist‐electric generator. *Advanced Functional Materials* **34**, 2311128 (2024).

13	Wang, H. *et al.* Bilayer of polyelectrolyte films for spontaneous power generation in air up to an integrated 1,000 V output. *Nature Nanotechnology* **16**, 811-819 (2021).

14	Huang, Z. *et al.* A hydrogel-based moist-electric generator with superior energy output and environmental adaptability. *Nano Energy* **126**, 109673 (2024).

15	Long, Y. *et al.* Moisture-induced autonomous surface potential oscillations for energy harvesting. *Nature communications* **12**, 5287 (2021).

16	Sun, Z. *et al.* Nanofiber fabric based ion-gradient-enhanced moist-electric generator with a sustained voltage output of 1.1 volts. *Materials Horizons* **8**, 2303-2309 (2021).

17	Zheng, S. *et al.* Continuous energy harvesting from ubiquitous humidity gradients using liquid‐infused nanofluidics. *Advanced Materials* **34**, 2106410 (2022).
